# An ATF3 Inducer Ameliorates Metabolic Dysfunction-Associated Steatotic Liver Disease Through the AMPK and PKA Pathways

**DOI:** 10.3390/ijms262411877

**Published:** 2025-12-09

**Authors:** Ching-Feng Cheng, Ruey-Bing Yang, Wen-Ting Chen, Jia-Fang Chung, Hui-Chen Ku

**Affiliations:** 1 Department of Pediatrics, Taipei Tzu Chi Hospital, Buddhist Tzu Chi Medical Foundation, New Taipei City 23142, Taiwan; chengcf@gms.tcu.edu.tw (C.-F.C.); cwenting54@gmail.com (W.-T.C.); diana88999@hotmail.com.tw (J.-F.C.); 2 Institute of Biomedical Sciences, Academia Sinica, Taipei 11529, Taiwan; rbyang@ibms.sinica.edu.tw; 3 Department of Pediatrics, Tzu Chi University, Hualien 97004, Taiwan

**Keywords:** activating transcription factor 3 inducer, MASLD, AMP-activated protein kinase, protein kinase A

## Abstract

Obesity is linked to metabolic dysfunction-associated steatotic liver disease (MASLD), but the molecular mechanisms and effective treatments remain unclear. This study investigated whether ST32db, an inducer of activating transcription factor 3 (ATF3), affects lipid metabolism in MASLD. An in vitro model was established involving the treatment of HepG2 cells with 1 mM oleic acid (OA) with or without 20 µM ST32db. In an in vivo model, C57BL/6 mice were fed a high-fat diet (HFD) for 18 weeks to induce obesity and treated or not with ST32db (1 mg kg^−1^). ST32db significantly decreased intracellular lipid accumulation in OA-treated HepG2 cells. In these cells, ST32db remarkably decreased mRNA and protein levels of adipogenesis- and lipogenesis-related genes and increased mRNA levels of adipose triglyceride lipase (ATGL), a lipolytic enzyme. In HFD-fed mice, the ST32db treatment significantly decreased the liver weight, serum triglycerides, and fat vacuole and triglyceride accumulation in the liver. Livers from these mice also showed significantly decreased CCAAT/enhancer-binding protein β mRNA and protein levels, increased ATF3 mRNA and protein and ATGL mRNA levels, and increased levels of phosphorylated AMP-activated protein kinase (AMPK) and protein kinase A (PKA). These findings suggest that ST32db may exert protective effects against MASLD through activating hepatic AMPK and PKA pathways.

## 1. Introduction

Metabolic dysfunction-associated steatotic liver disease (MASLD) is a common chronic liver disease worldwide with an estimated global prevalence of 30% to 40% [1,2,3]. The condition involves superfluous lipid accumulation in hepatocytes and liver tissues and is tightly associated with various chronic diseases, such as obesity, hyperlipidemia, and diabetes. A considerable proportion of people with MASLD (20–30%) develop nonalcoholic steatohepatitis, which can progress to advanced liver fibrosis, cirrhosis, and hepatocellular carcinoma. Although the pathogenesis of MASLD is not entirely understood, hepatic lipid metabolic disorders are likely crucial in the beginning stages and in its progression [4].

The liver has a central role in the modulation of energy metabolism and lipid transport. Evidence suggests that MASLD is induced by an imbalance of intracellular signaling pathways involved in triglyceride (TG) and fatty acid delivery, export, synthesis, or oxidation [5,6]. Lipogenesis and lipolysis are the main hepatic biological processes modulated by AMP-activated protein kinase (AMPK), a key intracellular energy sensor, and the cyclic AMP–protein kinase A (PKA) signaling pathway. Both signaling pathways represent promising therapeutic targets for the treatment of MASLD [7,8].

Activating transcription factor (ATF) 3 belongs to the ATF/cAMP response element-binding (CREB) family that binds to the cAMP response element in promoters with the consensus sequence TGACGTCA [9]. In adipocytes, ATF3 or ATF3 inducers inhibit adipogenesis and promote adipocyte browning [10]. In addition, hepatic ATF3 is important for the modulation of high-density lipoprotein metabolism, reversal of cholesterol transport, and atherosclerosis [11]. Xu et al. [12] found that the global or hepatocyte-specific loss of ATF3 exacerbates diet-induced steatohepatitis. They further reported that the hepatocyte-specific expression of ATF3 prevents diet-induced steatohepatitis via the induction of hepatic lipolysis and fatty acid oxidation and the suppression of inflammation and apoptosis.

Other ATF/CREB transcription factors, such as ATF4 and ATF6, also modulate hepatocyte function. In mice, ATF4 deficiency protects mice from hepatic steatosis induced by a high-fat diet (HFD) or by high-carbohydrate/high-fructose diets [13,14]. Moreover, a loss of ATF4 decreases TG accumulation in fructose-treated hepatocytes, whereas ATF4 overexpression enhances TG accumulation in HepG2 cells, indicating an effect of ATF4 on lipid metabolism in the liver [15]. ATF6 has been implicated in the regulation of lipogenesis. Whole-body ATF6 knockout mice show decreased mRNA levels of lipogenic genes, such as sterol regulatory element-binding protein 1c (SREBP1c) and peroxisome proliferator-activated receptor (PPAR)γ, and are resistant to HFD-induced MASLD [16]. In addition, Zeng et al. found that ATF6 suppresses lipogenesis and hepatic TG accumulation by recruiting the corepressor histone deacetylase 1 to the ATF6–SREBP2 complex bound to the sterol regulatory element within the promoters of lipogenic genes [17]. ATF6 is reported to alleviate liver lipogenesis and inflammation by stimulating sulfhydrylated sirtuin 1 via a cystathionine β synthetase/hydrogen sulfide synthesis pathway [18]. Conversely, the overexpression of active nuclear ATF6 promotes lipogenesis and hepatic steatosis in alcohol-treated zebrafish [19], even though the majority of evidence indicates that ATF6 suppresses lipid synthesis and alleviates hepatic steatosis.

Our previous work has shown that ST32db, a synthetic ATF3 inducer selected from the *ATF3* promoter screening platform, upregulates ATF3 expression in association with inhibited adipogenesis and lipogenesis and promoted adipocyte browning [10]. ST32db, which we have previously described in detail, also suppresses lipid droplet accumulation in 3T3-L1 adipocytes [10]. These findings have led to an interest in these pathways in the context of MASLD and other lipid disorders. Given the roles of lipogenesis and lipolysis in modulating lipid accumulation and the potential of ST32db in steatosis prevention, we hypothesized that ST32db would alleviate hepatic steatosis by inhibiting lipogenesis and enhancing lipolysis. Here, we investigated the mechanisms underlying improvements associated with ST32db in hepatic steatosis, using oleic acid (OA)-induced steatosis in HepG2 cells and HFD-induced obese mice as a model of MASLD.

## 2. Results

### 2.1. Effects of ST32db, an ATF3 Inducer, on the Viability of HepG2 Cells

**Figure 1:**
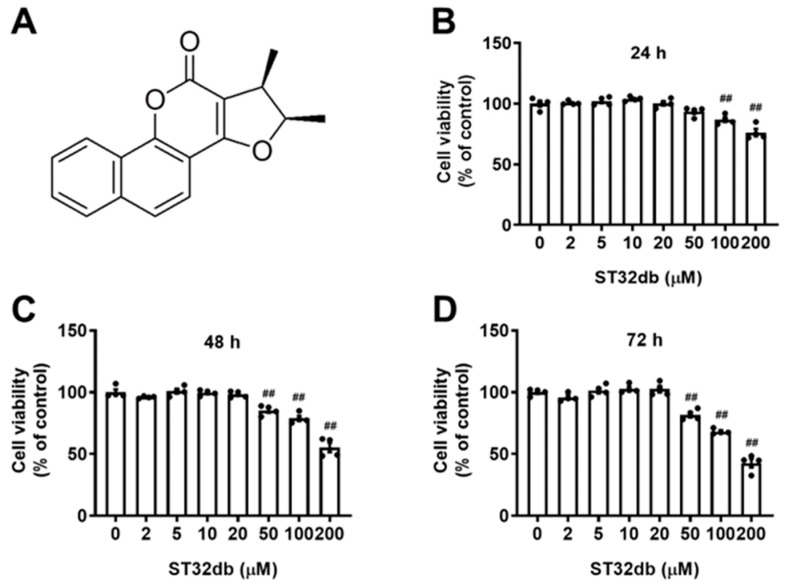
Effects of the ATF3 inducer, ST32db, on the viability of HepG2 cells. (**A**) The chemical structure of ST32db. (**B**–**D**) Cell viability was measured after the treatment with various concentrations of ST32db for 24 h, 48 h, and 72 h using a CCK8 assay. Data are mean ± SEM (*n* = 4) and analyzed by one-way ANOVA. ^##^ *p* < 0.01 compared to the control group.

To evaluate the cytotoxic effects of ST32db (Figure 1A) on HepG2 cells, we treated cells with various concentrations of ST32db for 24, 48, and 72 h. The cytotoxicity induced by ST32db was determined using a CCK8 assay. The data showed that the ST32db treatment (2–50 μM) for 24 h caused no cytotoxicity in the HepG2 cells (Figure 1B), although one of the higher concentrations of ST32db (100 μM) reduced the cell viability compared with control cells. HepG2 cells treated with ST32db (2–20 μM) for 48 or 72 h also showed no cytotoxic effects (Figure 1C,D). Based on these findings, we selected 20 and 50 μM ST32db for subsequent experiments.

### 2.2. ST32db Decreases Lipid Accumulation in OA-Treated HepG2 Cells

**Figure 2:**
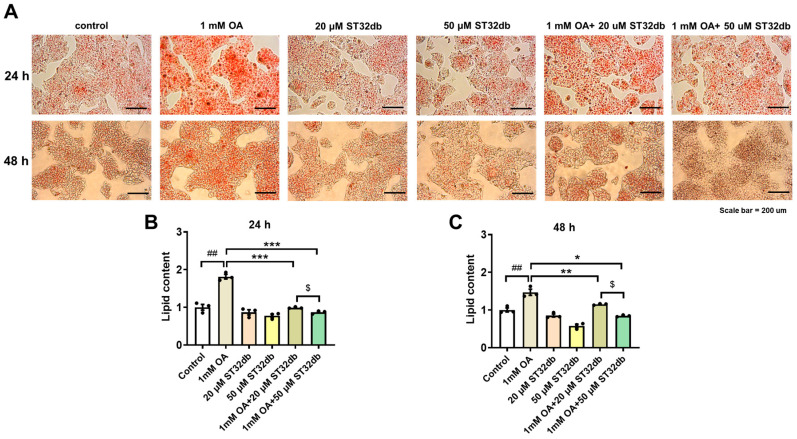
ST32db decreased lipid accumulation in oleic acid (OA)-treated HepG2 cells. Cells were treated with OA for 24 h, followed by ST32db treatment for 24 h or 48 h. (**A**) Representative images (200× magnification; scale bar, 200 µm) showing OA-induced lipid accumulation visualized by Oil Red O staining. (**B**,**C**) The relative quantification of lipid content. Data are mean ± SEM (*n* = 3) and analyzed by one-way ANOVA. ^##^ *p* < 0.01 compared to control group; * *p* < 0.05, ** *p* < 0.01, *** *p* < 0.001 compared to OA group; and ^$^ *p* < 0.05 compared to 1 mM OA+20 uM ST32db.

To determine the lipid-lowering effects of ST32db, HepG2 cells were treated with 1 mM OA for 24 h, followed by a 20 or 50 µM ST32db treatment for 24 h or 48 h. The intracellular lipid content was measured by Oil Red O staining. A microscopic examination showed that OA-treated HepG2 cells had serious steatosis compared with untreated control cells (Figure 2A). The lipid accumulation in the ST32db-treated cells was significantly decreased in a dose-dependent manner compared with the OA-treated cells (Figure 2B,C).

### 2.3. ST32db Regulates mRNA and Protein Levels of Genes Related to Adipogenesis, Lipogenesis, and Lipolysis in OA-Treated HepG2 Cells

**Figure 3:**
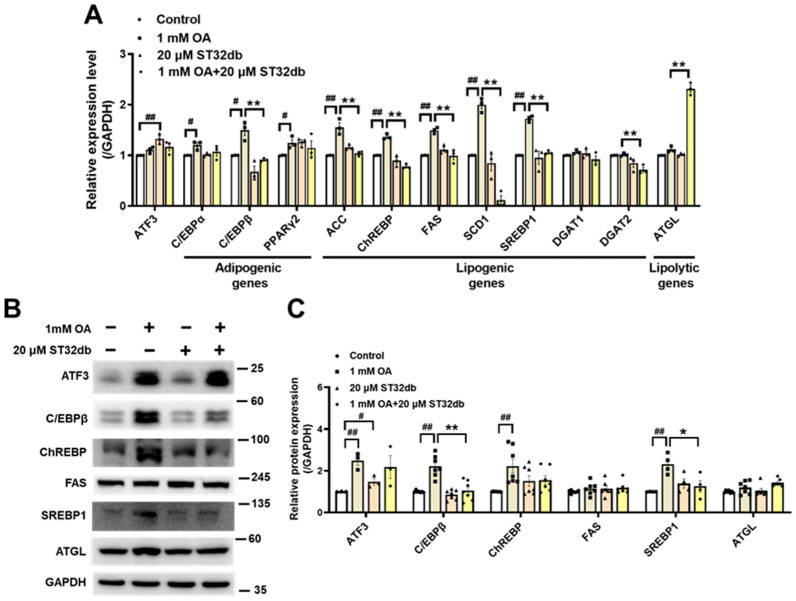
ST32db regulated adipogenesis-, lipogenesis- and lipolysis-related gene and protein levels in OA-treated HepG2 cells. (**A**) Real-time PCR analysis of mRNA levels of *ATF3*, adipogenic (*C/EBPα*, *C/EBPβ*, *PPARγ2)*, lipogenic (*ACC*, *ChREBP*, *FAS*, *SCD1*, *SREBP1*, *DGAT1*, and *DGAT2*), and lipolytic (*ATGL*) genes after 48 h co-treatment with OA and ST32db normalized to GAPDH and relative to control. (**B**) The levels of ATF3, C/EBPβ, ChREBP, FAS, SREBP1, and ATGL proteins were measured by Western blot. (**C**) Densitometry quantification of Western blot. Data are presented as mean ± SEM (*n* = 3~7) and analyzed by one-way ANOVA. ^#^ *p* < 0.05 and ^##^ *p* < 0.01 compared to control; * *p* < 0.05 and ** *p* < 0.01 compared to OA group.

To elucidate the mechanism of the ST32db inhibition of hepatic steatosis, we used a qPCR and Western blotting in HepG2 cells to analyze the expression of several key genes that affect adipogenesis (e.g., CCAAT/enhancer-binding protein α (C/EBPα), C/EBPβ, and PPARγ2), lipogenesis (e.g., acetyl-CoA carboxylase (ACC), carbohydrate-responsive element-binding protein (ChREBP), fatty acid synthase (FAS), stearoyl-CoA desaturase 1 (SCD1), SREBP1, diacylglycerol acyltransferase (DGAT)1 and DGAT2), and lipolysis (e.g., adipose triglyceride lipase (ATGL)). As shown in Figure 3A, the downregulation of *C/EBPβ*, *ACC*, *ChREBP*, *FAS*, *SCD1*, and *SREBP1* mRNA and upregulation of *ATGL* mRNA were observed in the ST32db-treated HepG2 cells compared with the OA-treated group. Consistent with changes in mRNA levels, protein levels of C/EBPβ and SREBP1 were markedly decreased in ST32db-treated HepG2 cells compared with OA-treated cells (Figure 3B,C). However, ATF3 mRNA and protein levels did not change in OA-exposed HepG2 cells treated with ST32db compared with OA controls (Figure 3). Additionally, a higher dose of ST32db (50 uM) showed similar effects on mRNA levels of these genes (Figure S1). These results suggest that ST32db alleviates hepatic steatosis by modulating the expression of genes involved in lipid synthesis and lipolysis in OA-induced HepG2 cells.

### 2.4. ST32db Ameliorates HFD-Induced Hepatic Steatosis

**Figure 4:**
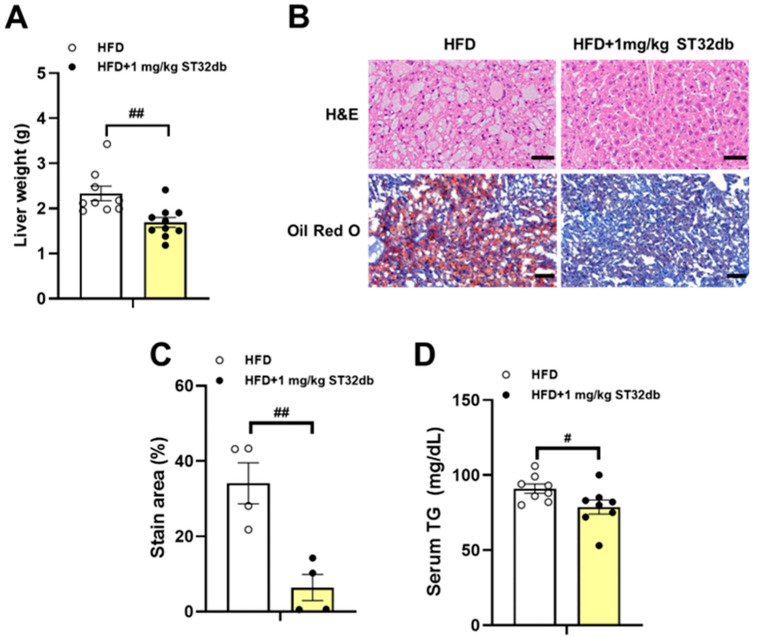
Inhibitory effect of ST32db on liver weight, hepatic TG accumulation, and serum TG levels in HFD-fed mice. Analysis of wild-type mice fed a HFD for 18 weeks with or without intraperitoneal injection of ST32db at a dose of 1 mg kg^−1^ twice weekly. (**A**) Liver weight (*n* = 9~10). (**B**) Hematoxylin and eosin (H&E) and Oil Red O staining of liver sections (original magnification × 200, *n* = 4). (**C**) Quantification of Oil Red O staining. (**D**) Serum TG levels (*n* = 8). Scale bar in H&E and Oil Red O =  50 and 100 µm, respectively. Data are presented as mean ± SEM and analyzed by one-way ANOVA. ^#^ *p* < 0.05 and ^##^ *p* < 0.01 compared to HFD group.

In our previous study [10], a ST32db treatment (1 mg kg^−1^ twice weekly) for 18 weeks significantly reduced the body weight gain in HFD-induced obese mice compared with controls but did not affect food intake. In the present study, we asked whether the ST32db treatment could alleviate fatty livers in this mouse model of obesity and found that the ST32db treatment substantially reduced the liver weight compared with controls (Figure 4A). In our histopathological assessment of the fat accumulation in livers from these animals, we found that the ST32db treatment substantially decreased the size of the fat droplets in hepatocytes (Figure 4B). A quantification analysis revealed that the ST32db administration led to significantly decreased lipid accumulation (Figure 4B,C) and decreased serum TG levels (Figure 4D). In addition, we found that administering a higher dose of ST32db (10 mg/kg twice weekly) did not further reduce the body weight or total fat mass compared with either the control group or the lower dose group (1 mg/kg twice weekly) (Figure S2).

### 2.5. ST32db Increases ATF3 mRNA Levels but Decreases Adipogenesis- and Lipogenesis-Related mRNA in Mouse Liver

**Figure 5:**
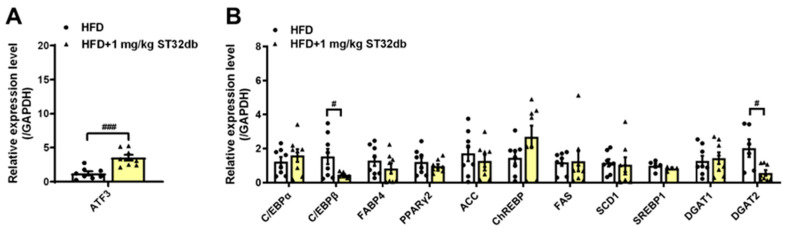
ST32db increased ATF3 mRNA levels but decreased the mRNA levels of adipogenesis- and lipogenesis-related genes in the liver of HFD-fed mice. (**A**) The mRNA levels of ATF3 and (**B**) adipogenesis- and lipogenesis-related gene expression were measured by real-time PCR analysis. Data are presented as mean ± SEM (*n* = 8) and analyzed by one-way ANOVA. ^#^ *p* < 0.05 and ^###^ *p* < 0.001 compared to HFD group.

To elucidate the molecular mechanism of the effects of ST32db on MASLD, we examined mRNA levels associated with ATF3 and genes involved in hepatic lipogenesis. We found that compared with HFD-only controls, ST32db upregulated *ATF3* mRNA levels in the liver (Figure 5A) and that mRNA levels of *C/EBPβ* and *DGAT2* were downregulated in ST32db-treated livers (Figure 5B), similar to the effect of ST32db on HepG2 cells. However, ST32db exerted a weaker suppressive effect on the SREBP1 mRNA expression compared with HFD-only controls (*p* = 0.31). These results suggest that ST32db can alleviate hepatic steatosis in MASLD by upregulating ATF3 expression and suppressing de novo lipogenesis in the liver.

### 2.6. ST32db Promotes Lipolysis in the Mouse Liver

**Figure 6:**
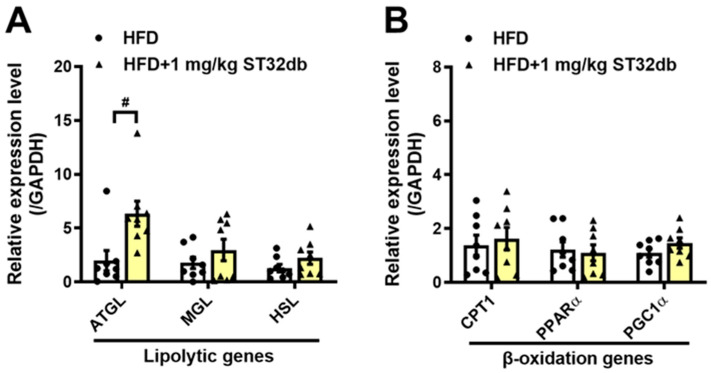
ST32db upregulated the mRNA levels of lipolytic genes in the liver of HFD-fed mice. (**A**) The mRNA levels of lipolytic genes, including ATGL, MGL, and HSL and (**B**) β-oxidation genes, including Cpt1, PPARα, and PGC1α, were measured by a real-time PCR analysis. Data are presented as the mean ± SEM (*n* = 8) and analyzed by one-way ANOVA. ^#^ *p* < 0.05 compared to the HFD group.

We further examined the lipolytic activity of ST32db by analyzing the expression of key enzymes in the hepatic TG metabolism in mice, including ATGL, monoacylglycerol lipase, and hormone-sensitive lipase, and the β-oxidation enzymes carnitine palmitoyl transferase 1, PPARα, and PPARγ coactivator 1α. Compared with controls, *ATGL* mRNA levels in the liver increased after the treatment with ST32db (Figure 6A), but mRNA levels of β-oxidation enzymes seemed to be unaffected (Figure 6B). Our data indicate that the increased lipolytic ability of ST32db depends primarily on the upregulated expression of *ATGL*, similar to the effect of ST32db on HepG2 cells.

### 2.7. ST32db Increases Levels of ATF3, Phospho-PKA, and Phospho-AMPK Proteins but Decreases C/EBPβ Protein Levels in Mouse Liver

**Figure 7:**
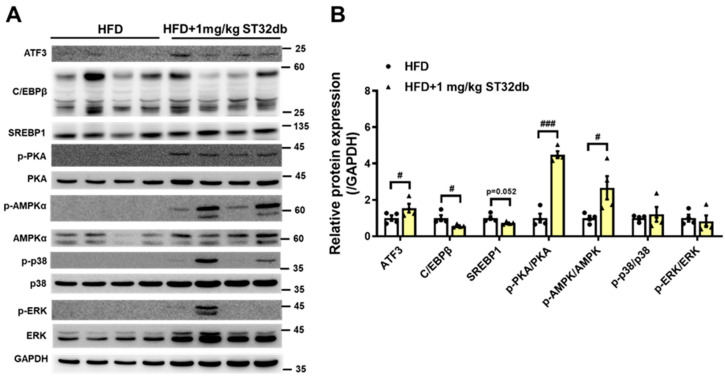
(**A**) Western blot and (**B**) densitometry quantification showing effects of ST32db on protein expression of ATF3; C/EBPβ; and phosphorylated form of AMPK, PKA, p38, and ERK in the liver of HFD-fed mice. The protein levels were measured by Western blot. Data are presented as mean ± SEM (*n* = 4) and analyzed by one-way ANOVA. ^#^ *p* < 0.05 and ^###^ *p* < 0.001 compared to HFD group.

To clarify the molecular mechanism underlying the effects of ST32db on MASLD, including suppressed lipid accumulation in the liver and improved TG levels, we examined the expression of proteins involved in hepatic lipogenesis (C/EBPβ and SREBP1) and lipolysis (phosphorylation [p] of AMPK, PKA, p38, and ERK) (Figure 7). Consistent with the in vitro results, levels of C/EBPβ protein were downregulated in ST32db-treated livers compared with the control mouse liver. ST32db showed a trend toward decreasing SREBP1 protein levels relative to the control group (*p* = 0.052). In addition, ST32db significantly increased ATF3, p-AMPK, and p-PKA protein levels compared with controls. These results suggest that ST32db can ameliorate hepatic steatosis in MASLD by enhancing lipolysis and suppressing de novo lipogenesis through the activation of the AMPK and PKA pathways in the liver.

## 3. Discussion

ATF3 is a crucial metabolic modulator with important roles in regulating metabolism, immunity, and oncogenesis [20]. We previously demonstrated that the ATF3 inducer ST32db may have beneficial effects in obesity and metabolic dyshomeostasis [10]. Because MASLD is a related condition, here we sought to investigate the potential therapeutic effects of ST32db on HFD-induced MASLD and to explore the molecular mechanisms involved. MASLD is characterized by an enormous aggregation of fat in the liver, resulting from the conversion of superfluous free fatty acids into TGs and their accumulation in hepatocytes [21]. To assess the protective impacts and molecular mechanisms of action of ST32db in MASLD, we used in vitro (OA-treated HepG2 cells) and in vivo (HFD-induced obese mice) models.

Although OA is an unsaturated fatty acid with reported antioxidative and anti-inflammatory properties, it is widely used to establish in vitro models of hepatic steatosis because it effectively promotes neutral lipid accumulation while causing minimal cytotoxicity. The biological actions of OA may vary depending on its concentration and the cellular context. In this study, 1 mM OA was applied to HepG2 cells to induce steatosis [22], leading to a significantly increased accumulation of lipid droplets. In these cells, the ST32db treatment exerted a suppressive effect on this OA-induced hepatic steatosis. In addition, ST32db significantly decreased the OA-induced mRNA levels of *C/EBPβ*, *ACC*, *ChREBP*, *FAS*, *SCD1*, *SREBP1*, and *DGAT2*. At the protein level, the ST32db treatment in these cells led to a significantly dampened production of OA-induced C/EBPβ and SREBP1, transcriptional factors that modulate downstream genes involved in de novo lipogenesis, such as those encoding ACC, FAS, and SCD1 [23]. Furthermore, we found that ST32db reduced hepatic steatosis by decreasing the expression of de novo lipogenesis genes and by upregulating lipolysis-related *ATGL* mRNA. The physiological function of ATGL may not be limited to adipose tissue and could involve many other tissues, such as the liver [24,25], although it is expressed at low levels in hepatocytes. Liver-specific ATGL overexpression decreases hepatic steatosis and slightly improves liver insulin sensitivity [26], and hepatic ATGL expression is positively associated with fatty acid β-oxidation [27]. In addition, patients with MASLD have decreased hepatic ATGL levels [28]. Although ST32db is an ATF3 inducer, it did not increase ATF3 mRNA or protein levels in OA-treated cells. This lack of upregulation may reflect the influence of OA-induced lipogenic signaling, which could dampen ST32db-mediated ATF3 induction through feedback regulation or altered cellular stress response pathways.

In the in vivo experiment, we investigated whether ST32db improves hepatic steatosis in HFD-induced MASLD. We found that ST32db significantly decreased the liver weight in the HFD-fed obese mice. Consistent with the in vitro results, ST32db significantly slowed the progression of HFD-induced MASLD, as evidenced by fewer and smaller lipid vacuoles on histological examination. In addition, serum TG levels declined significantly with the ST32db treatment.

Also consistent with the in vitro findings, ST32db decreased C/EBPβ mRNA and protein levels in the mouse liver in our in vivo model. We further observed the downregulation of *DGAT2* mRNA and the upregulation of *ATGL* mRNA in ST32db-treated mouse livers compared to controls, along with an increased expression of ATF3, phospho-PKA, and phospho-AMPK proteins in ST32db-treated livers. A potential limitation of this study is the absence of a normal diet control group, which would have provided an additional baseline for the comparison of metabolic and histological parameters.

The results of the animal and cell experiments in the present study together indicate that ST32db modulates the expression of genes involved in lipogenesis and lipolysis, resulting in the amelioration of hepatic steatosis. PKA plays an important role in the modulation of lipid metabolism, and the dysmodulation of its signaling is linked to pathogenic mechanisms underlying several metabolic diseases, such as MASLD [29]. PKA is an upstream signaling molecule of AMPK, which it can activate by phosphorylating Thr 172 [30]. To date, no available drugs target PKA or AMPK to treat lipid-related metabolic syndromes in hepatocytes.

**Figure 8:**
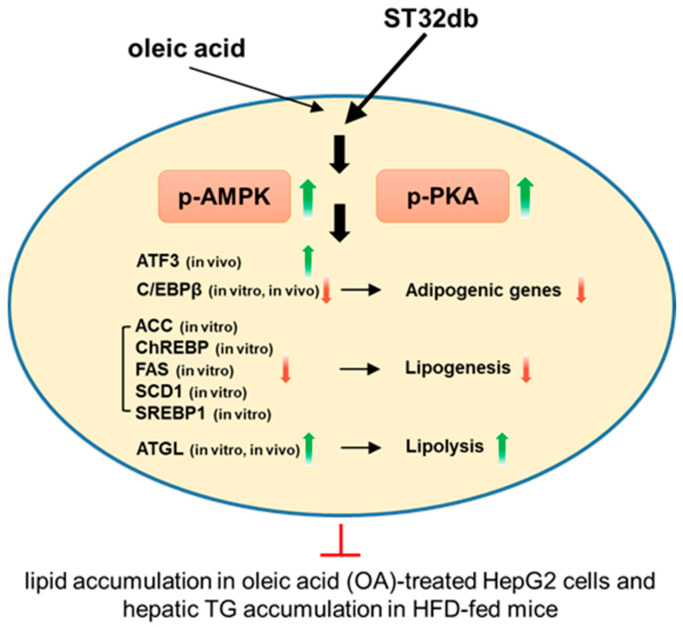
A schematic diagram of the molecular mechanisms of the anti-MASLD effect of the ATF3 inducer ST32db. ST32db protects against diet-induced MASLD by inhibiting lipogenesis and enhancing lipolysis via the activation of AMPK and PKA pathways. Green upward arrows indicate increases; red downward arrows indicate decreases; red T-shaped lines indicate inhibition.

The activation of AMPK, an important modulator of numerous metabolic pathways, inhibits hepatic lipogenesis and increases fatty acid oxidation [31]. AMPK also inhibits adipogenesis-related proteins such as C/EBPβ and PPARγ [32]. C/EBPβ can induce the expression of PPARγ and C/EBPα, which subsequently promote the expression of adipocyte-specific genes. Our qPCR and Western blot results indicate that the OA treatment increased the C/EBPβ expression in HepG2 cells and that ST32db dampened this effect. These results suggest that the suppression of the adipogenic protein expression may underlie the decline in lipid accumulation following ST32db exposure. Our in vivo and in vitro experiments showed that ST32db modulates the expression of genes involved in adipogenesis, lipogenesis, and lipolysis, leading to the amelioration of hepatic steatosis (Figure 8).

## 4. Materials and Methods

### 4.1. Materials and Reagents

Fetal bovine serum and Dulbecco’s modified eagle medium (DMEM) were purchased from Gibco Life Technology (Grand Island, NY, USA). Penicillin, streptomycin, oleic acid, and Oil Red O staining powder were purchased from Sigma-Aldrich (St. Louis, MO, USA). A cell counting kit (CCK-8) was purchased from Abcam (Cambridge, UK).

### 4.2. Cell Culture

Human hepatoma HepG2 cells were obtained from ATCC (HB-8065, ATCC, Manassas, VA, USA). HepG2 cells were cultured in DMEM supplemented with 10% fetal bovine serum and 1% penicillin–streptomycin in a humidified incubator with 5% CO_2_ at 37 °C.

### 4.3. Analysis of Cell Viability

HepG2 cells were cultured in 96-well plates at 5 × 10^3^ per well and treated with ST32db (at a concentration of 2, 5, 10, 20, 50, 100, and 200 μM) for 24, 48, and 72 h. Then, cell viability was analyzed by CCK-8 assay.

### 4.4. Oil Red O Staining

Lipid droplet accumulation in HepG2 cells was measured using Oil Red O staining. Cells were seeded in 6-well plates (4 × 10^5^ cells/well) and incubated with 1 mM oleic acid (OA) for 24 h. After washing with PBS, cells were treated with ST32db (20 or 50 μM) for 24 or 48 h. Cells were then fixed with 10% formalin for 1 h, stained with Oil Red O for 1 h, and washed with distilled water. The dye was eluted with 100% isopropanol, and absorbance was measured at 500 nm.

### 4.5. Real-Time Quantitative PCR

Total RNA from cultured cells or liver tissues was extracted using TRIzol reagent (Invitrogen, Carlsbad, CA, USA) and reverse-transcribed into cDNA using the iScript cDNA Synthesis Kit (Bio-Rad, Hercules, CA, USA). Real-time quantitative PCR (qPCR) was performed on an ABI PRISM 7700 Sequence Detection System (Applied Biosystems, Foster City, CA, USA) using SYBR Green (Bio-Rad). Primer sequences are listed in Table 1. Relative gene expression levels were normalized to GAPDH, and fold changes were calculated using the 2^−ΔΔCT^ method.

### 4.6. Western Blot Analysis

Cells were lysed in RIPA buffer, and liver tissues (200 mg) were homogenized using a tissue ruptor (Qiagen, Hilden, Germany). Lysates were centrifuged at 14,000 rpm for 15 min at 4 °C, and protein concentrations were measured by the Bradford assay. Proteins were separated by SDS-PAGE, transferred to PVDF membranes (Merck Millipore, Burlington, MA, USA), and probed with antibodies, including AMPKα (#2603, Cell Signaling Technology), Phospho-AMPKα (Thr172) (#2535, Cell Signaling Technology, Danvers, MA, USA), p44/42 MAPK (ERK1/2) (#4695, Cell Signaling Technology), Phospho-p44/42 MAPK (ERK1/2) (#9101, Cell Signaling Technology), PKA C-α (#4782, Cell Signaling Technology), Phospho-PKA C (Thr197) (#4781, Cell Signaling Technology), p38 MAPK (#9212, Cell Signaling Technology), Phospho-p38 MAPK (Thr180/Tyr182) (#9211, Cell Signaling Technology), ATF3 (#ab207434, Abcam, Cambridge, UK), ChREBP (#81958, Abcam), C/EBPβ (sc-7962, Santa Cruz, Dallas, TX, USA), fatty acid synthase (#3180, Cell Signaling Technology), SREBP1 (14088-1-AP, Proteintech, Rosemont, IL, USA), ATGL (#2138, Cell Signaling Technology), and GAPDH (#2118, Cell Signaling Technology). Secondary antibodies (1:1000 in 5% milk) included anti-rabbit IgG-HRP (#7074) and anti-mouse IgG-HRP (#7076, Cell Signaling Technology). Protein bands were detected using a chemiluminescence reagent (PerkinElmer, Shelton, CT, USA) and quantified with ImageJ software version 1.53t (National Institutes of Health, Bethesda, MD, USA).

### 4.7. Animal Studies

Six-week-old male C57BL/6 mice (National Center for Biomodels, Taipei, Taiwan) were fed a high-fat diet (HFD, 45% kcal from fat) with or without intraperitoneal ST32db for 18 weeks to assess its effects on fatty liver. Body weight and food intake were recorded weekly. All procedures were performed according to the protocols approved by the Institutional Animal Care and Utilization Committee, Academia Sinica, Taipei, Taiwan. The HFD alone was set as control group. HFD: *n* = 9; HFD + 1 mg·kg^−1^ ST32db: *n* = 10; HFD + 10 mg·kg^−1^ ST32db: *n* = 7.

### 4.8. Histopathological Assessments of Livers

The liver samples of mice were fixed in 4% paraformaldehyde at room temperature for 24 h and embedded in paraffin and sectioned into 5 μm thick sections. Liver slices were stained with hematoxylin–eosin (H&E) and Oil Red O for histological examinations.

### 4.9. Statistical Analyses

Values are expressed as means ± SEM from at least three experiments. The data were analyzed using one-way ANOVA, followed by Tukey’s test. A value of *p* < 0.05 was considered statistically significant.

## 5. Conclusions

The ST32db treatment suppressed lipid accumulation and steatosis in vitro and in an in vivo MASLD model. It also significantly decreased the liver weight and liver steatosis in HFD-fed obese mice. Results from both models implicated the modulation of lipid metabolism, including the inhibition of C/EBPβ, ACC, ChREBP, FAS, SCD1, SREBP1, and DGAT2 expression and the stimulation of ATGL, p-PKA, and p-AMPK production, in the effects of the ST32db treatment. These pathways may thus underlie the protective effects of ST32db against MASLD.

## Figures and Tables

**Table 1:** Primer sequences for real-time PCR.

Gene Name	Forward Primer 5′-3′	Reverse Primer 5′-3′	Gene Name	Forward Primer 5′-3′	Reverse Primer 5′-3′
*mATF3*	CTCCTGGGTCACTGGTATTTG	CCGATGGCAGAGGTGTTTAT	*mPPARα*	GTCCTCAGTGCTTCCAGAGG	GGTCACCTACGAGTGGCATT
*mC/EBPα*	GTAACCTTGTGCCTTGGATACT	GGAAGCAGGAATCCTCCAAATA	*mGAPDH*	GGAGCCAAACGGGTCATCATCTC	GAGGGGCCATCCACAGTCTTCT
*mC/EBPβ*	CTTGATGCAATCCGGATCAAAC	CCCGCAGGAACATCTTTAAGT	*hATF3*	CTGGAAAGTGTGAATGCTGAAC	ATTCTGAGCCCGGACAATAC
*mPPARγ2*	CTGGCCTCCCTGATGAATAAAG	AGGCTCCATAAAGTCACCAAAG	*hC/EBPα*	GAAGTCGGTGGACAAGAACA	TCATTGTCACTGGTCAGCTC
*mFABP4*	GCTCCTCCTCGAAGGTTTAC	CCCACTCCCACTTCTTTCAT	*hC/EBPβ*	CGCGACAAGGCCAAGAT	GCTGCTCCACCTTCTTCTG
*mACC*	TGATGGTGGCCTGCTCTTGTCTTA	CAGCAAACACATGTCCGCCATCTT	*hPPARγ2*	GCCTGCATCTCCACCTTATTA	ATCTCCACAGACACGACATTC
*mFAS*	AGACCCGAACTCCAAGTTATTC	GCAGCTCCTTGTATACTTCTCC	*hACC*	CAGAAGTGACAGACTACAGG	ATCCATGGCTTCCAGGAGTA
*mDGAT1*	GGCCTTACTGGTTGAGTCTATC	GTTGACATCCCGGTAGGAATAA	*hFAS*	TGGTCACGGACGATGACCGTCG	GCGGCAGTACCCATTCCCCGC
*mDGAT2*	GAAGGGCTTCTCTTCTCTTCAC	CTTTCTCCCAACGCCTCATAA	*hDGAT1*	CTGGTCCAGTCTTGGGGTCT	ACCAAGCTGGATAGATGGGG
*mSCD1*	TGGGTTGGCTGCTTGTG	GCGTGGGCAGGATGAAG	*hDGAT2*	CTGGAGAACCTCATCAAGTATGG	CAAAGACATTGGCCGCAATAA
*mChREBP*	TGTTCAGCATCCTCATCCGACCTT	TGAGTTGGCGAAGGGAATTCAGGA	*hSCD1*	ACAACTACCACCACTCCTTTC	GGAGACTTTCTTCCGGTCATAG
*mATGL*	CATCCGTGGCTGTCTACTAAAG	GACGTTCTCTCCGTCTGAAAC	*hChREBP*	GGAAGAATTTCAAAGGCCTCAAG	CTCTTCCTCCGCTTCACATAC
*mHSL*	GGACGGTCCTAGGTTTGAATAC	GATGGGAAGGTCTGTGGTTAC	*hSREBP1*	GAGCCATGGATTGCACTTTC	AGCATAGGGTGGGTCAAATAG
*mMGL*	GACAGAAAGAGTGTGGGAAGAG	CTGAGCACAGTAGTCTGGAATG	*hATGL*	AACACCAGCATCCAGTTCA	TATCCCTGCTTGCACATCTC
*mPGC1α*	CTAGCCATGGATGGCCTATTT	GTCTCGACACGGAGAGTTAAAG	*hGAPDH*	GGTGTGAACCATGAGAAGTATGA	GAGTCCTTCCACGATACCAAAG
*mCPT1*	GAAGTGTCGGCAGACCTATTT	GTCCTCCTCTCTATATCCCTGTT			

## Data Availability

The original contributions presented in this study are included in the article. Further inquiries can be directed to the corresponding author.

## Supplementary Materials

The following supporting information can be downloaded at https://www.mdpi.com/article/10.3390/ijms262411877/s1.

## Abbreviations

ACC	Acetyl-CoA carboxylase
AMPK	AMP-activated protein kinase
ATF	Activating transcription factor
ATGL	Adipose triglyceride lipase
C/EBPα	CCAAT/enhancer-binding protein α
ChREBP	Carbohydrate-responsive element-binding protein
CREB	cAMP response element-binding protein
DGAT1	Diacylglycerol acyltransferase 1
DMEM	Dulbecco’s modified eagle medium
FAS	Fatty acid synthase
H&E	Hematoxylin–eosin
HFD	High-fat diet
MASLD	Metabolic dysfunction-associated steatotic liver disease
OA	Oleic acid
PKA	Protein kinase A
PPAR	Peroxisome proliferator-activated receptor
SCD1	Stearoyl-CoA desaturase 1
SREBP1	Sterol regulatory element binding protein 1
TG	Triglyceride
