# Supplementary material for: An ATF3 Inducer Ameliorates Metabolic Dysfunction-Associated Steatotic Liver Disease Through the AMPK and PKA Pathways
Source: Int J Mol Sci. 2025 Dec 9;26(24):11877. doi: 10.3390/ijms262411877 (PMC12732720; doi:10.3390/ijms262411877)
Supplement: Supplementary file 1 [file ijms-26-11877-s001.zip › ijms-3958129-supplementary.pdf]

Supplementary Materials

Manuscript: An ATF3 inducer ameliorates metabolic dysfunction-associated steatotic liver disease through the AMPK and PKA pathways

Authors: Ching-Feng Cheng, Ruey-Bing Yang, Wen-Ting Chen, Jia-Fang Chung, Hui-Chen Ku

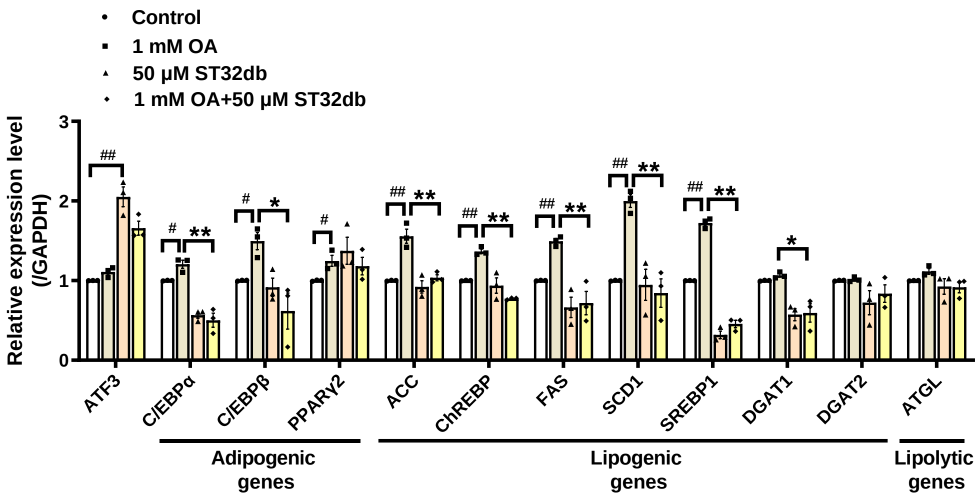

Figure S1. ST32db regulated mRNA expression of adipogenesis-, lipogenesis- and lipolysis-related genes in OA-treated HepG2 cells. Real-time PCR analysis of mRNA levels of *ATF3*, adipogenic (*C/EBPα, C/EBPβ, PPARγ2)*, lipogenic (*ACC, ChREBP, FAS, SCD1, SREBP1, DGAT1, and DGAT2*) and lipolytic (*ATGL*) genes after 48 h co-treatment with OA and ST32db normalized to GAPDH and relative to control. Data are presented as mean ± SEM (n = 3) and analyzed by One-way ANOVA. #p < 0.05, ##p < 0.01 compared to control; *p < 0.05, **p < 0.01 compared to OA group.

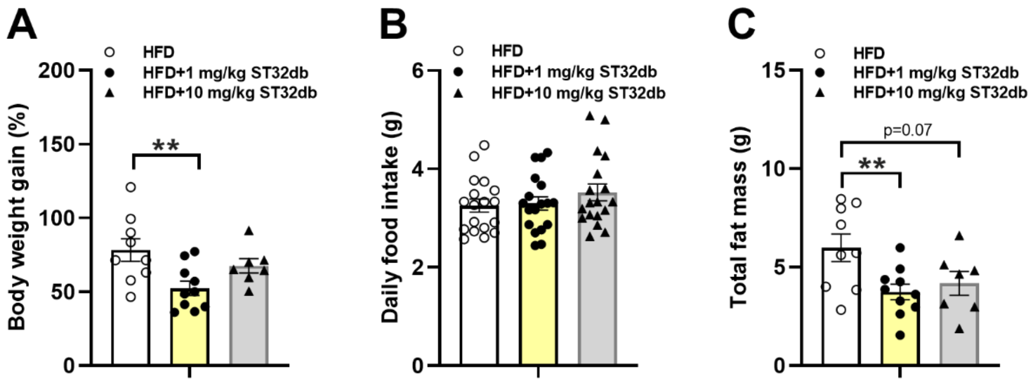

Figure S2. ST32db attenuated HFD-induced obesity. Wild-type mice were fed HFD for 18 weeks and received intraperitoneal injections of ST32db (1 mg kg^{-1} or 10 mg kg^{-1}) twice weekly.

(A) Body weight. (B) Food intake. (C) Total fat mass. Data are presented as mean ± SEM (HFD: n = 9; HFD + 1 $mg \cdot kg^{-1}$ ST32db: n = 10; HFD + 10 $mg \cdot kg^{-1}$ ST32db: n = 7) and analyzed by one-way ANOVA. $^{**}p < 0.01$ compared to HFD control group.
